# Medical–Bioethical Mediation as a Potential Governance Innovation for Sustainable, Efficient, and Resilient Healthcare Systems

**DOI:** 10.3390/healthcare14142082

**Published:** 2026-07-12

**Authors:** Olympia Lioupi, Katarzyna Czabanowska, Łukasz Balwicki, Polychronis Kostoulas

**Affiliations:** 1 Faculty of Public & One Health, School of Health Sciences, University of Thessaly, 43100 Karditsa, Greece; olioupi@uth.gr; 2 Department of International Health, Care and Public Health Research Institute (CAPHRI), 6229 GT Maastricht, The Netherlands; kasia.czabanowska@maastrichtuniversity.nl; 3 Department of Public Health and Social Medicine, Faculty of Health Sciences, Medical University of Gdańsk, 80-211 Gdańsk, Poland; balwicki@gumed.edu.pl

**Keywords:** medical–bioethical mediation, healthcare conflict, health governance, public health ethics, health system resilience

## Abstract

**Highlights:**

**What are the main findings?**

- The paper proposes medical–bioethical mediation as a population-level governance framework for recurring ethical and institutional conflict in healthcare systems.
- The framework links conflict identification, stakeholder mapping, ethical clarification, mediated dialogue, negotiated commitments, implementation, and feedback-based learning.

**What are the implications of the main findings?**

- Medical–bioethical mediation may support more constructive handling of resource allocation, inter-organizational coordination, public health restrictions, and system reform.
- Its possible contribution to sustainability, efficiency, and resilience remains hypothesis-generating and requires empirical testing.

**Abstract:**

Background/Objectives: Healthcare systems increasingly operate through dense networks of clinicians, managers, public health authorities, institutions, and communities. Disagreement is no longer confined to bedside decisions; it also affects coordination, legitimacy, and implementation at system level. Bioethics, administrative governance, and consultation processes address important parts of this problem, but they do not by themselves provide a dedicated process for handling persistent disagreement, mistrust, and competing values across actors and institutions. This paper proposes medical–bioethical mediation as a potential governance innovation for sustainable, efficient, and resilient healthcare systems. Methods: This is a conceptual framework paper based on an integrative synthesis of selected literature in clinical bioethics mediation, medical mediation, public health ethics, deliberative and participatory governance, trust, inter-organizational coordination, health governance, and health system resilience. It does not report empirical research or a systematic review. Results: The proposed population-level framework is organized around seven linked components: conflict identification, stakeholder mapping, ethical issue clarification, mediation, negotiated solutions, implementation, and feedback and learning. These components form three broader moments: diagnosis, mediated deliberation, and implementation with learning. The framework identifies mechanisms through which mediation may affect health system governance, including conflict transformation, structured ethical deliberation, participation, trust-building, coordination, and adaptive learning. Illustrative scenarios include resource allocation, inter-organizational coordination, public health restrictions, vaccine mistrust, and system reform. The framework is proposed as a hypothesis-generating governance model rather than as a tested intervention. Conclusions: Medical–bioethical mediation should be considered beyond the bedside as a possible system-level governance approach. Its potential value lies in helping health systems manage recurring ethical and institutional conflict more constructively and may strengthen the conditions for legitimacy, coordination, implementation, and learning. Its potential contribution should be understood as plausible but unproven at population level and should be evaluated through pilot implementation, economic analysis, and comparative case research.

## 1. Introduction

Contemporary healthcare involves conflicts that extend well beyond the bedside disputes that traditionally anchor discussions of mediation. Health systems are shaped by interdependence among clinicians, managers, regulators, insurers, public health authorities, and communities. Decisions rarely unfold within a single institution. More often, they depend on relationships across governance levels, service networks, and communities [1,2]. Disputes over evidence-based priority setting, resource allocation, service reorganization, inter-hospital coordination, public health restrictions, crisis response, and community acceptance of contested policies are intensified by fragmentation, competing institutional interests, mistrust, crisis pressure, and divergent views of evidence and fairness. These conflicts are often structural rather than episodic. Coordination problems in multilevel healthcare networks and tensions in innovation processes demonstrate this pattern [3,4]. Work on trust, crisis response, and evidence–policy conflict shows why resistance may follow even technically informed decisions. Communities or institutions may experience such decisions as unfair, opaque, or imposed [5,6,7,8]. In such settings, disagreement is a recurring feature of how complex health systems make and implement decisions.

Existing approaches address important parts of this problem, but not the problem as a whole. Bioethics provides normative guidance where fairness, proportionality, and legitimacy are at stake. Ethical frameworks clarify what should count in a decision, but they do not by themselves organize conflict among actors who interpret those criteria differently or no longer trust the process [9]. The distinction is important. Fiester’s critique of ethics consultation points to the same limit when consultation is expected to do the work of mediation [10,11]. Public health ethics adds essential values for population-level conflict. The APHA Public Health Code of Ethics emphasizes professionalism and trust, health justice and equity, solidarity, human rights and civil liberties, inclusivity, and engagement [12]. European health system principles similarly emphasize universality, quality, safety, equity, solidarity, evidence-informed care, and ethical practice. They also emphasize patient involvement and confidentiality [13]. These values support defensible decisions, but they still require processes capable of handling disagreement when values, risks, and obligations conflict.

Governance offers authority, mandates, and administrative process. Yet it often lacks a dedicated mechanism for transforming persistent conflict. Deliberative and policy dialogue processes are important in contested health decisions, and participation and public reasoning matter for legitimacy [14,15,16]. The remaining gap is not another forum for consultation alone. It is a process that can move actors from positional standoff toward negotiated engagement. Bioethics mediation offers a basis for this move by combining ethical clarification with relational process, shared problem definition, and negotiated follow-through [17,18,19]. Medical–bioethical mediation therefore differs from leadership, health diplomacy, change management, ethics consultation, complaint handling, and patient liaison services; its focus is the structured handling of ethically charged conflict where relationships, legitimacy, and implementation are at stake.

This paper proposes medical–bioethical mediation as a potential governance innovation for sustainable, efficient, and resilient healthcare systems. It develops a conceptual framework for extending mediation beyond individual clinical disputes to organizational, inter-institutional, and population-level health governance. Section 2 presents medical–bioethical mediation as a governance innovation, Section 3 sets out the proposed population-level framework, Section 4 explains its core mechanisms, Section 5 presents illustrative application scenarios, and Section 6 outlines implementation architecture. Section 7 discusses expected benefits, safeguards, limitations, and the research and policy agenda.

### Conceptual Approach and Framework Development

This manuscript develops a conceptual framework; it does not report empirical research or a systematic review. The framework was developed through an integrative conceptual synthesis of selected literature in clinical bioethics mediation, medical mediation, public health ethics, deliberative and participatory governance, health system resilience, trust, inter-organizational coordination, and health governance. These domains were selected because they correspond to the main functions needed to handle ethically charged conflict at system level: ethical clarification, conflict transformation, stakeholder participation, legitimacy, coordination, implementation, and institutional learning.

The literature was identified purposively rather than through a systematic search strategy. Sources were selected to represent established concepts in bioethics mediation and healthcare conflict resolution, together with adjacent governance studies relevant to population-level decision-making under disagreement. The aim was not to review all of the literature on mediation or health governance exhaustively, but to integrate concepts that help explain how ethically charged conflict can be recognized, convened, deliberated, negotiated, implemented, and translated into learning.

The seven components of the framework were derived by combining core elements of mediation practice with governance functions required for health system conflict management. Conflict identification and stakeholder mapping reflect the diagnostic phase—whether mediation is appropriate and who must be included. Ethical issue clarification and mediation/deliberation reflect the process phase, in which values, interests, responsibilities, and constraints are made explicit; negotiated solutions, implementation, and feedback/learning reflect the governance phase, in which dialogue is translated into commitments, follow-through, and institutional adaptation. The framework is therefore deductive in drawing on existing theory and practice, and synthetic in integrating these elements into a proposed population-level governance framework.

Accordingly, the framework should be read as a conceptual proposal rather than as a validated intervention. The manuscript distinguishes between what is established in clinical bioethics mediation and medical dispute mediation, what is extrapolated from governance, public health ethics, and resilience literature, and what is newly proposed here as a population-level extension. Empirical testing, pilot implementation, economic evaluation, and comparative case research are needed before claims can be made about effectiveness at system level.

## 2. Medical–Bioethical Mediation as a Governance Innovation

### 2.1. Clinical Roots and Conceptual Distinction

Medical–bioethical mediation is a structured process for handling ethically charged healthcare conflict through facilitated dialogue. A trained and credible facilitator helps affected actors identify the conflict, clarify the ethical stakes, hear one another’s reasons, and negotiate workable next steps. This understanding is grounded in clinical bioethics mediation and recent work on medical–bioethical mediation [10,11,18,19]. Transformative mediation further clarifies why mediation works on interactional dynamics rather than settlement alone [17]. The relevant contrast is with adjudication, escalation, or managerial fiat. Mediation does not decide for the parties; it creates a disciplined process in which contested meanings, interests, and responsibilities can be worked through.

Because this framework overlaps with several established health system practices, Table 1 clarifies how medical–bioethical mediation differs from adjacent approaches.

The framework proposed here does not replace these approaches. It identifies a narrower but important function that no single adjacent practice fully captures: the transformation of ethically charged conflict into negotiated and implementable commitments, with explicit attention to legitimacy, stakeholder inclusion, institutional accountability, and learning. In some settings, medical–bioethical mediation may be embedded within ethics services, complaint systems, public health agencies, or governance bodies. Its distinctiveness depends less on organizational location than on whether the process combines ethical clarification, impartial facilitation, negotiated movement, implementation, and feedback.

This distinction matters because healthcare conflict is rarely only informational. It often reflects moral pluralism, competing interpretations of fairness, and disagreement over what counts as a legitimate process [9,20]. Medical–bioethical mediation is therefore not interchangeable with ethics consultation, governance, leadership, health diplomacy, change management, complaint handling, or patient liaison services. These functions may clarify principles, set direction, negotiate policy, manage implementation, or receive grievances. Mediation has a more specific role: conflict transformation when actors must continue to act together despite disagreement.

Medical–bioethical mediation has clinical roots. It is used in bedside disputes, end-of-life decisions, communication breakdowns, and conflicts among patients, families, clinicians, and institutions [19,21,22,23]. These cases establish the features that travel into the broader argument: neutrality, structured dialogue, attention to values, and preservation of workable relationships where decisions cannot simply be deferred. They also mark the boundary of the concept. A process that simply communicates a decision, collects complaints, or secures compliance would not count as medical–bioethical mediation in the sense used here. The defining element is the organized movement from conflict recognition to ethical clarification, dialogue, negotiation, and follow-through.

### 2.2. From Clinical Mediation to Population-Level Governance

The population-level claim changes the scale and purpose of mediation. The conflict is no longer confined to a single episode of care. It may involve organizations, regulators, professional groups, public authorities, communities, and policy systems; it may concern allocation, reform, coordination, preparedness, or legitimacy. Integrated care is linked to sustained dialogue across professional and organizational boundaries [24]. Policy dialogue is also presented as a multistakeholder governance tool [16]. Intersectoral collaboration and multilevel healthcare networks further show how coordination problems are often distributed across institutions rather than contained within a single clinical encounter [4,25]. These features support the move from case-based mediation toward system-oriented conflict governance.

Current evidence does not justify treating population-level medical–bioethical mediation as a mature or already institutionalized framework. It does justify treating it as an emerging extension of clinical mediation into settings where actors must negotiate values, evidence, and implementation under conditions of interdependence. Active health governance is relevant here. It frames health decision-making as coordination among governments, organizations, and publics, rather than as a series of isolated institutional decisions [26].

In institutional terms, mediation becomes a governance layer rather than only a case-level technique. Existing institutional and third-party mediation models show that conflict-handling can be formalized within healthcare settings [22,27]. They do not yet show the full population-level form proposed here. The broader governance literature points in the same direction: health systems need processes that handle disagreement without reducing it to administrative compliance [14,16,26]. Work on local health governance and integrated care further emphasizes collaborative action. It does so across institutional boundaries [2,24]. The legitimacy of such arrangements depends not only on outcomes, but also on whether affected actors regard the process as credible, fair, and open to meaningful participation [28].

This framing also explains why the framework is not simply a larger version of bedside mediation. Population-level mediation would need to work across institutional mandates, public authority, community voice, and professional accountability. Its distinctive contribution would be to create a process through which these actors can clarify the conflict, make value claims explicit, and develop workable commitments without pretending that consensus already exists.

### 2.3. Contribution to Sustainable, Efficient, and Resilient Health Systems

The performance contribution of mediation should be understood as conditional. Mediation is not useful in every case, and dialogue is not always faster or preferable to other modes of decision-making. Its potential value lies in situations where recurring conflict, mistrust, or implementation failure cannot be resolved by formal authority alone. In those settings, mediation may strengthen institutional conditions. Those conditions can make sustainability, efficiency, and resilience more attainable.

For sustainability, the central issue is whether healthcare institutions remain socially and politically workable over time. The people-centered framework directs attention to governance arrangements that remain responsive to populations [1], while legitimacy is a condition of durable governance [28]. Unresolved conflict can erode that durability by accumulating mistrust and implementation fatigue. Studies of integrated care show how collaborative processes can support implementation across diverse stakeholders [24], and work on fragile and conflict-affected settings reinforces that durable systems depend on the capacity to govern complexity under pressure [29]. Mediation may contribute by reducing the buildup of relational and ethical tensions that weaken system functioning.

Here, the sustainability claim is institutional rather than idealistic. It does not assume that all disagreement can be resolved. The point is that repeated avoidance or suppression of disagreement can damage the relationships and procedures on which continuing cooperation and public legitimacy depend. Nor is the argument that mediation is always quicker or cheaper. The stronger claim is that badly managed conflict creates downstream costs through escalation, litigation, duplication, delayed implementation, and lost opportunities for safety learning [30,31]. Gordon [24], Van der Weert [4], and Robert [16] all point to the efficiency value of coordination across actors whose priorities do not naturally align, while Haring [3] shows that unresolved tensions in healthcare innovation can hinder implementation. Baltussen’s [14] deliberative framework is relevant here because contested decisions require explicit process design. Mediation extends that insight by addressing the relational breakdowns that often prevent decision criteria from being applied effectively.

For resilience, mediation is most relevant when systems must adapt under stress. Resilience is linked to governance capacities that remain effective during disruption [32,33,34], and is especially visible in fragile or conflict-affected settings [29,35]. Crises intensify disputes over priorities, evidence, organizational responsibility, and public trust. Work on trust and mistrust during crisis [8] makes it difficult to separate technical response capacity from relational legitimacy. Mediation does not itself produce resilience, but it may support mechanisms associated with resilience: communication across conflict lines, containment of escalation, coordination under pressure, learning from recurrent disputes, and negotiated movement when values and interests diverge.

The three domains are connected. Sustainability concerns the durability of governance arrangements, efficiency concerns the avoidable costs of unmanaged conflict, and resilience concerns adaptive capacity under stress. Mediation can plausibly contribute to each by making conflict visible, structured, and usable for institutional learning. This is not a claim that mediation guarantees sustainability, efficiency, or resilience. It is a hypothesis that mediation may strengthen several governance capacities through which those aims become more plausible in complex health systems and contested institutional settings. The design question is how medical–bioethical mediation should be organized at population level.

## 3. Proposed Population-Level Framework

### 3.1. Core Components

The proposed framework has seven linked components: conflict identification, stakeholder mapping, ethical issue clarification, mediation process, negotiated solutions, implementation, and feedback and learning. These components form three broader moments: diagnosis, mediated deliberation, and implementation with learning. The sequence should be read as a coherent governance process rather than as a fixed protocol. It begins by defining the conflict, moves through structured engagement and negotiation, and ends with follow-through and institutional learning. The framework draws most directly on healthcare mediation literature, particularly work on medical–bioethical mediation and structured clinical conflict resolution [19,36,37], while its extension toward population-level governance is supported by adjacent work on deliberative priority-setting, policy dialogue, public health ethics, and stakeholder participation [9,14,15,16]. The aim is not to create a parallel bureaucracy, but to specify how ethically charged conflict can be recognized, convened, worked through, and translated into institutional action.

To make the framework more operational, Table 2 summarizes the purpose, actors, outputs, and possible evaluation indicators associated with each component.

These indicators are illustrative rather than prescriptive. Their role is to show how future pilot studies could move beyond simple agreement rates and examine whether mediation affects trust, perceived legitimacy, implementation adherence, recurrence of conflict, time to resolution, stakeholder experience, and institutional learning. The choice of indicators should depend on the level of application, the type of conflict, and the governance context.

The framework is also multi-level. At the micro level, it builds on clinical mediation work addressing communication breakdown, ethical disagreement, end-of-life decision-making, and continuing care relationships [19,21,38]. At the meso level, it extends this logic to hospitals, provider networks, and regional systems. In these settings, interdependence, uneven authority, and implementation conflict often require structured negotiation rather than ordinary managerial coordination [4,24]. At the macro level, it speaks to health governance settings involving ministries, public health authorities, communities, and policy actors. These actors must work through contested priorities in complex multi-actor systems [1,16,26]. Importantly, the framework does not assume that bedside mediation can simply be scaled up unchanged; rather, it argues that the underlying logic of structured, ethically attentive dialogue can be adapted across levels.

Diagnosis includes conflict identification and stakeholder mapping. Conflict identification marks the point at which disagreement requires structured engagement rather than routine management in governance settings. Here, conflict recognition is treated as a practical governance skill [36,39]. Stakeholder mapping then asks who must be included for the process to be legitimate and useful, including actors who may be less visible but materially affected by the outcome. The quality of this process depends on who is recognized as relevant and who is excluded too early [15,16]. Exclusion can occur too early. This diagnostic moment matters because mediation can otherwise reproduce the same blind spots that contributed to the dispute.

Mediated deliberation includes ethical issue clarification and the mediation process itself. Ethical issue clarification prevents the framework from becoming a purely managerial coordination exercise by naming the fairness, legitimacy, proportionality, and responsibility questions at stake; contested decisions need justificatory reasoning as well as administrative authority [9]. The mediation process then creates the setting in which these values can be discussed alongside interests, constraints, and possible movement. This link between ethical reflection and relational process is central to bioethics mediation [10,11,18].

Implementation with learning includes negotiated solutions, implementation, and feedback. Negotiated solutions may be substantive agreements, procedural commitments, revised implementation terms, shared monitoring arrangements, or clarified responsibilities. Implementation is included because agreement without follow-through has little governance value; governance processes can fail when agreement is not translated into workable coordination [16,24]. Feedback and learning complete the framework because population-level conflict is rarely a one-off event. Adaptive systems are linked with the capacity to learn from stress rather than merely absorb it [32,34]. Each mediated conflict may therefore help the system learn how to handle future disagreement more credibly.

### 3.2. Actors Involved

Participation depends on the conflict and the level of application. At the clinical level, relevant actors may include mediators, clinicians, patients, families, and institutional representatives. At organizational and population level, managers, policy officials, public health authorities, ethicists, legal advisors, frontline professionals, and community representatives may also be needed. Population health governance often depends on interactions across sectors and organizations rather than within one administrative chain [16,25]. This plurality matters because many health system conflicts persist when one actor group assumes that it alone defines the problem correctly, while work on deliberative democracy reminds us that publics can contribute to policy reasoning rather than only receive decisions [40]. A parallel organizational argument is that implementation may improve when stakeholders are engaged as contributors to problem-solving rather than treated as obstacles [24]. The mediator’s role is not to replace expertise, but to structure interaction among different forms of expertise, authority, and lived experience.

### 3.3. Process Flow

Operationally, the framework moves from trigger to assessment, mediation, deliberation, negotiation, agreement, and monitoring. A trigger indicates that routine governance is no longer sufficient. Assessment determines whether mediation is appropriate, which actors must be included, and which ethical or procedural issues require clarification before formal engagement begins. Mediation opens the structured dialogue, while deliberation asks participants to articulate reasons, values, and constraints rather than only preferences. Negotiation searches for acceptable movement across those positions. Agreement should be treated as a practical and provisional achievement, not necessarily as deep consensus. Monitoring then asks whether commitments are implemented and whether the process produces learning for future conflicts.

**Figure 1:**
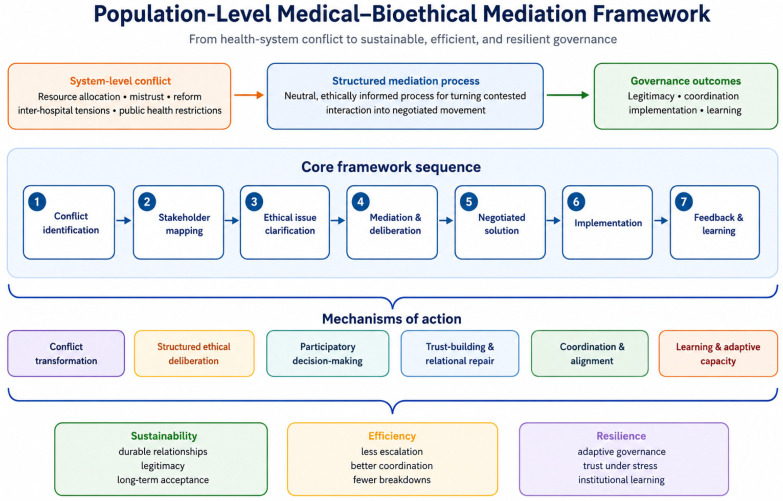
Population-level medical–bioethical mediation framework. The figure presents the proposed framework as a sequence moving from system-level conflict through structured mediation, core process components, mechanisms of action, and expected governance contributions to sustainability, efficiency, and resilience.

This process flow expresses the framework’s core claim. Population-level mediation is not a single conversation inserted into governance, but a way of organizing ethically contested interaction over time. Current evidence does not establish population-level medical–bioethical mediation as a mature framework; it supports the framework as a conceptual argument grounded in prior work on mediation, health governance, public health ethics, deliberation, trust, and resilience. It also clarifies why the seven components should be read together rather than as isolated tools or disconnected procedural steps. Figure 1 summarizes the proposed population-level medical–bioethical mediation framework.

## 4. Core Mechanisms of Action

The mechanisms of action explain how mediation may affect health system governance once a structured process is in motion. They do not restate the seven components. Instead, they identify how mediation may change the handling of conflict by reframing interaction, organizing ethical disagreement, widening participation, rebuilding enough trust for cooperation, improving coordination, and generating learning. Each mechanism is conditional, context-sensitive, and process-dependent rather than automatic or guaranteed by institutional design.

### 4.1. Conflict Transformation and Ethical Deliberation

The first mechanism is conflict transformation, since mediation can alter interactional dynamics rather than merely produce settlement [17]. Fiester and Bergman [10,11,36] add a healthcare-specific basis by linking moral impasse, conflict-source identification, and clinical mediation. At population level, this matters. Actors may be locked into positional reasoning and may interpret disagreement as obstruction. Tensions in healthcare innovation often reflect contradictions between organizational logics, professional norms, and system pressures, not only bad faith or technical error [3], and mediation can make those contradictions discussable and may shift a dispute from zero-sum confrontation toward a shared account of what must be resolved.

The second mechanism is structured ethical deliberation, which within bioethics mediation is distinct from ordinary discussion or ethics consultation alone [10,11,18]. Mediation requires participants to give reasons, consider the distributive and moral implications of their positions, and respond openly to competing principles. This is especially important in public health, where fairness, justification, and procedural reason-giving are central to legitimate decisions [9], and where contested choices need deliberative structures that make trade-offs visible [14]. The APHA Code similarly treats ethical analysis as a process that considers values, facts, uncertainty, and obligations together [12].

These two mechanisms are linked. Conflict transformation without ethical deliberation could become a managerial exercise in calming dispute without addressing the underlying value conflict. Ethical deliberation without mediation can remain too abstract. This is especially true when actors distrust one another or when institutional stakes are immediate. The distinctive mechanism lies in their combination: mediation keeps ethical reasoning attached to the relationships, constraints, and implementation choices that make disagreement consequential.

### 4.2. Participation, Trust, and Coordination

Participation matters because it changes who is allowed to define the problem and what counts as an acceptable response. In public and health system decisions, affected groups are not only recipients of policy. They can also contribute to policy reasoning [40]. Process design determines whether that contribution is real: who is invited, which claims are recognized, and how disagreement is handled [15]. Work on policy dialogue and integrated care similarly shows that stakeholders are more useful when engaged as contributors to problem-solving. They should not be treated as obstacles to be managed [16,24]. This is also consistent with the APHA Code’s emphasis on inclusivity, engagement, dialogue, mutual respect, reciprocity, and trust [12].

Trust is closely linked to participation. Mediation does not manufacture trust, nor does it require goodwill at the outset. Its contribution is more modest and more practical: it creates conditions in which actors can test one another’s claims more credibly than they can under unilateral assertion or institutional command. This institutional trust function is visible in mediation and communication resolution contexts [22,23,41]. It is also important in public health crises, where trust and mistrust shape health behaviour and cooperation [8], and where trust in health organizations depends on how authority is experienced in practice [7]. People-centred crisis response makes the same point by linking trust to communication, engagement, and institutional responsiveness [41].

Coordination connects participation and trust to implementation. Multilevel healthcare networks depend on alignment among organizations that remain formally distinct [4]. Similar lessons emerge from integrated care, policy dialogue, and intersectoral governance, where cooperation requires structured interaction rather than general appeals to collaboration [16,24,25]. Mediation can help convert diffuse disagreement into explicit negotiation over roles, responsibilities, constraints, and feasible terms of cooperation. Its value is to surface relational and normative barriers before they harden into implementation failure.

These mechanisms reinforce one another: participation brings otherwise invisible concerns into the process, and trust-building makes it possible to examine those concerns without immediate escalation. Coordination translates clarified concerns into workable responsibilities and action. Mediation is therefore not only a forum for voice; it is a process through which voice, recognition, and negotiated responsibility may support more stable cooperation.

### 4.3. Learning and Adaptive Capacity

Learning and adaptive capacity form the final mechanism. Population-level mediation is relevant not only because it may help resolve a particular dispute in a given setting. It can also reveal how similar conflicts arise and recur. Adaptation is a core feature of resilient systems [32,34]. Health systems learn under pressure only when crisis experience is translated into changes in process and coordination [33]. Mediation can support that translation by making conflict legible, rather than allowing it to disappear into blame and fatigue.

A mediated dispute may expose recurring fault lines in governance design, communication, stakeholder inclusion, and implementation. Related lessons can be drawn from mediation and communication resolution contexts linked to patient safety and institutional learning [30,41]. More broadly, tensions in healthcare systems are often patterned rather than exceptional [3]. This is especially visible in fragile settings, where adaptive capacity depends on learning from conflict rather than merely surviving it [29,35]. Mediation should therefore be valued not only for the agreements it may produce, but also for the feedback it generates about how the system itself is functioning.

Learning is not an additional benefit that appears after mediation has ended; it is part of the process. When mediation identifies recurring triggers, excluded actors, unclear mandates, or implementation gaps, the system gains information about how future disputes might be prevented or governed with less damage to legitimacy and cooperation.

## 5. Illustrative Application Scenarios

Table 3 summarizes five illustrative scenarios. These are not empirical case studies. They show where the framework could potentially operate and what kinds of conflict it is designed to address. Across these scenarios, mediation is used as a governance process rather than as a substitute for policy choice, technical expertise, or legal authority. The examples are deliberately schematic: their purpose is to show how the framework might apply to recurring types of system-level conflict without claiming that these settings already represent established population-level medical–bioethical mediation.

In vaccine hesitancy, the framework would address mistrust among communities, public health authorities, and healthcare institutions. It would not treat hesitancy as an individual knowledge deficit. Trust-related work suggests that mistrust is not corrected by information alone [7,8], while people-centered crisis response supports a shift from one-way messaging toward structured engagement [41]. Mediation would help clarify whether the dispute concerns safety perceptions, exclusion from decision-making, inconsistent communication, or deeper institutional distrust. In disputes over public health restrictions, the central issues are often proportionality, liberty, collective protection, and legitimacy, and such conflicts cannot be reduced to technical administration because they involve moral disagreement about acceptable burdens and public justification [9,20]. Mediation would not decide in advance which side is correct; it would make the reasons for restriction, objection, and possible adjustment more explicit.

Resource allocation is a strong illustrative domain because triage, benefit design, and trade-off decisions are ethically charged and socially contested [14,42]. Mediation would not displace expert judgment or planning, but it could organize disagreement over fairness, priority, and justification. It could also support procedural commitments about how decisions are communicated, reviewed, and monitored. That function becomes especially important in crisis conditions, where scarcity intensifies and weaknesses in governance design become more visible [33,34].

Inter-hospital conflicts illustrate the coordination function of mediation. Interorganizational healthcare networks are often prone to coordination problems, especially when referral pathways, regional capacity, and institutional responsibilities are poorly aligned [4]. Work on integrated care and collaborative governance similarly shows why structured cooperation matters when service delivery depends on multiple institutions [24,43]. Formalized healthcare mediation mechanisms in institutional settings provide useful analogues. They should not, however, be treated as full implementations of the population-level framework proposed in this paper [27,44]. Here, mediation is not private dispute settlement between institutions. It is a process for making roles, expectations, incentives, and monitoring arrangements explicit before organizational tensions harden into implementation failure [3].

System reform conflicts show the framework at its widest scale. Reform is rarely only technical; it usually involves disagreement over evidence, priorities, legitimacy, and whose interests are advanced [1,5,6,26]. Collaborative governance becomes especially important when reforms cut across organizations and local systems [2,16]. Mediation would not remove politics from reform or replace democratic accountability. Its contribution would be to provide a structured process for handling political and institutional disagreement so that reform is less likely to collapse into deadlock, symbolic consultation, or unilateral implementation.

### Worked Example: Vaccine Mistrust and Public Health Communication Conflict

Vaccine mistrust provides a useful worked example because the conflict is rarely only informational. It may involve safety concerns, inconsistent communication, prior negative experiences with institutions, perceived exclusion from decision-making, misinformation, professional disagreement, and wider mistrust of public authority. In this setting, ordinary public health communication may be necessary but insufficient if affected communities experience the policy process as imposed or dismissive.

The first component, conflict identification, would involve recognizing several warning signs. Low uptake, repeated public objections, professional disagreement, or escalating mistrust may indicate a governance conflict rather than a simple knowledge deficit. The trigger for mediation could come from several sources. These include a public health authority, local healthcare organization, community representative, professional association, or regional governance body. The initial question would not be whether one party is correct, but whether the conflict has become sufficiently recurrent, ethically charged, and implementation-limiting to require structured engagement.

The second component, stakeholder mapping, would identify the actors whose participation is needed for the process to be credible and useful. The relevant actors may be diverse. They may include public health officials, primary care clinicians, school or community representatives, patient groups, local leaders, representatives of vulnerable groups, communication experts, and, where appropriate, legal or ethics advisors. Stakeholder selection should be transparent and should explicitly consider groups affected by the policy but often underrepresented in formal consultation.

The third component, ethical issue clarification, would make explicit the values and trade-offs at stake. These may include autonomy, collective protection, proportionality, equity, transparency, protection of vulnerable persons, and fair distribution of burdens. This step prevents the process from being reduced to persuasion or message correction. It also helps participants distinguish factual disagreement, uncertainty, value conflict, and mistrust.

The fourth component, mediation and deliberation, would provide a setting in which participants can explain concerns, challenge assumptions, hear public health reasoning, and explore possible movement. The mediator would not decide vaccination policy or replace scientific expertise. Instead, the mediator would facilitate a disciplined process in which public health objectives, community concerns, institutional responsibilities, and possible adjustments are discussed together.

The fifth component, negotiated solutions, may produce substantive or procedural commitments. These could include revised communication materials, locally trusted messengers, clearer explanation of adverse event monitoring, community feedback channels, targeted support for access barriers, agreed timelines for review, or transparent procedures for responding to new evidence. The aim is not necessarily full consensus, but a more legitimate and workable pathway for implementation.

The sixth component, implementation, would specify who is responsible for each commitment, what resources are required, and how progress will be monitored. Without this step, mediation risks becoming symbolic dialogue. Implementation may involve public health agencies, local providers, community organizations, and communication teams.

The seventh component, feedback and learning, would assess whether the process changed the trajectory of the conflict. Possible indicators include stakeholder perceptions of fairness, trust in the process, uptake patterns, recurrence of complaints, misinformation dynamics, participation of previously excluded groups, and the responsiveness of public health authorities to concerns raised during the process. The learning function matters because vaccine mistrust often recurs across programmes and crises. A mediated process should therefore generate institutional knowledge about how future public health conflicts can be handled earlier and more credibly.

This worked example illustrates the distinctive contribution of population-level medical–bioethical mediation. It does not replace public health authority, scientific evidence, legal responsibility, or communication strategy. Rather, it creates a structured process through which ethically charged disagreement, mistrust, and implementation barriers can be clarified, negotiated, and translated into accountable action.

## 6. Implementation Architecture

### 6.1. Institutional Location and Triggers

Population-level medical–bioethical mediation needs an identifiable institutional location, but not a single fixed organizational form. It may operate as a dedicated mediation service for complex health system disputes, as an embedded governance function within public health agencies, hospital networks, regional bodies, complaint pathways, patient-safety programs, or ethics structures, or as a competency set for trained health system actors. Existing governance sites already carry much of the practical burden of collaboration and may therefore provide plausible anchors for mediation capacity [2,24]. Where disputes involve public authorities or intersectoral conflict, regional health bodies, public health agencies, or independent units may be more credible, especially where institutional or third-party mediation models already show how conflict-handling can be formalized in healthcare settings [22,23,44].

The central requirement is functional independence. If mediation is too closely tied to one actor’s authority structure, it may be perceived as a disguised management tool rather than as a legitimate governance process. Institutional location should therefore fit the scale and composition of the conflict [26]. Activation should also be selective, with recurring triggers such as policy deadlock, persistent mistrust, implementation failure, repeated interorganizational conflict, and ethically charged disagreement under uncertainty. Conflict recognition is itself a practical skill in healthcare mediation and conflict management [36,39], while innovation tensions and politics–evidence conflict may signal that ordinary governance is no longer sufficient [3,5,6].

Mediation should be used when ordinary governance remains formally active but no longer produces workable implementation. The relevant threshold is whether structured engagement could plausibly change the trajectory of a conflict that threatens trust, coordination, or follow-through.

### 6.2. Training, Competencies, and Safeguards

Implementation depends on trained actors who can combine mediation skill with health system literacy. Evidence on formal competency models for population-level medical–bioethical mediators remains limited. Healthcare-specific mediation capacity is needed [19,45], but training must also be translated into practice rather than treated as a one-off educational intervention [46]. Effective practice would require ethical reasoning, facilitation skill, and stakeholder analysis. It would also require the ability to work across organizational boundaries and power asymmetries [9,15,24]. The role cannot be reduced either to generic mediation training or to subject matter expertise alone; it requires deliberate role design as well as training.

Safeguards are equally important. Fairness, transparency, and legitimacy are procedural conditions for credible mediation processes, not optional additions to them [9,14]. Inclusion must also be designed rather than assumed [15]. Minimal safeguards should include mandate clarity, transparent process design, defensible inclusion criteria, protection against coercive participation, and monitoring of outcomes after agreement. The APHA Code supports this safeguard logic. It emphasizes transparency, accountability, participation, least restrictive action, and protection of vulnerable groups in public health decision-making [12]. Mediation will be viable only if the process is seen as credible, accountable, and resistant to capture by stronger participants [28].

### 6.3. Neutrality, Power Asymmetry, and Functional Independence

At population level, mediator neutrality requires special attention because public authorities, healthcare institutions, or professional bodies may themselves be parties to the conflict. In such cases, neutrality cannot mean complete social or political detachment from the health system. A more realistic and defensible standard is functional independence, procedural impartiality, transparency of mandate, and protection from unilateral control by any one actor.

This matters especially where power asymmetries are strong, such as disputes involving ministries of health, regional authorities, hospital networks, professional groups, or marginalized communities. If the mediator is appointed, funded, or hosted by an institution perceived as a party to the dispute, the process may deepen rather than reduce mistrust. Safeguards are therefore required. These may include arm’s-length appointment procedures, multi-party oversight, public terms of reference, conflict-of-interest declarations, transparent criteria for stakeholder inclusion, independent documentation of process outcomes, and the possibility of appointing external mediators in high-stakes disputes.

Neutrality should also be understood as process neutrality rather than moral indifference. The mediator should not impose the substantive outcome, but should actively protect fairness of participation, clarity of ethical reasoning, recognition of affected groups, and resistance to coercion or symbolic consultation. This distinction matters because population-level mediation must remain ethically attentive while avoiding capture by stronger institutional actors.

### 6.4. Agility During Crises and Emergencies

The framework should not be interpreted as requiring a full multi-stage process in every situation. Acute emergencies may require rapid public health or administrative action, and mediation should not delay decisions where delay would create serious harm. In crisis settings, the framework may need to operate in a compressed form, focusing on rapid conflict assessment, ethical clarification, targeted stakeholder engagement, and short-cycle feedback.

The role of mediation during crises is not to replace emergency authority or technical command structures, but to improve the legitimacy, proportionality, communication, and adaptability of decisions as the response evolves. During an acute outbreak, for example, a full mediation process may be inappropriate before urgent containment measures are taken. Structured engagement can still be used rapidly to clarify contested burdens, identify excluded groups, explain proportionality, revise implementation details, and create feedback channels. In this form, the framework may support crisis governance without becoming logistically paralyzing.

### 6.5. Implementation Analogues and Practical Entry Points

Population-level medical–bioethical mediation is not yet an established practice, but adjacent practices suggest feasible entry points. Medical dispute mediation and third-party mediation mechanisms in hospitals show that conflict-handling can be formalized within healthcare organizations rather than left to informal negotiation [22,23,27,44]. These examples should be read as analogues, not as full implementations of the framework.

Other adjacent practices point in the same direction. Communication-and-resolution programmes for medical error disclosure link disclosure, negotiation, and institutional learning [31,47]. Patient liaison, complaint, or ombuds services may provide practical sites where mediation capacity could be housed or connected, provided they have procedural independence and staff trained to mediate rather than only receive complaints. Policy dialogue and public deliberation offer related governance processes for stakeholder engagement [15,16]. These analogues support feasibility modestly: they identify possible entry points, but not completed examples of population-level medical–bioethical mediation.

The practical implication is incremental development. Health systems could begin by strengthening mediation capacity within existing governance, safety, ethics, and complaint structures, while specifying when conflicts should be escalated to a more independent process.

## 7. Discussion

### 7.1. Conceptual Contribution

This discussion is organized around the conceptual contribution of the framework, the practical conditions for responsible use, the risks and limitations of population-level mediation, and the research and policy agenda required before implementation at scale. The main contribution is to treat recurring health system conflict as a governance problem rather than as an episodic failure of communication or compliance. The claim is cautious. Potential benefits are most plausible where legitimacy and trust are threatened by disagreement in contested health governance settings. Procedural quality is closely linked to the acceptability of difficult decisions [1,14,28]. Mistrust is often intensified when actors experience decisions as exclusionary or misrecognizing [7,8,41]. In adjacent institutional mediation and communication resolution contexts, structured processes can support relational repair and learning [22,23,47]. The point is practical. Cooperation may also improve when actors have a process for negotiating coordination rather than merely absorbing directives [4,16,24]. The contribution is therefore practical as well as conceptual. Mediation may help systems convert recurrent conflict into clarification, negotiation, and follow-through. This is especially relevant where technically defensible decisions fail because actors dispute the process, the distribution of burdens, or the credibility of the institution making the decision.

### 7.2. Practical Conditions for Responsible Use

Responsible use depends on design conditions: a credible mandate, functional independence, transparent procedures, defensible inclusion criteria, and integration with implementation and feedback. Contested governance processes work better when their scope is explicit. Their connection to decision-making must also be clear [14,16]. Leadership support, organizational commitment, and procedural legitimacy are also necessary if the process is to be trusted [24,28]. The framework should be used where ordinary governance remains formally active but no longer produces cooperation, legitimacy, or workable implementation; it should not become a universal first response or a symbolic consultation exercise. These conditions also protect the framework from being confused with consensus-seeking for its own sake. Responsible mediation must allow disagreement to remain visible while still producing clearer responsibilities and next steps. Follow-through is crucial, because a process that generates dialogue without implementation will quickly lose credibility.

### 7.3. Cost, Feasibility, and Economic Evaluation

Cost and feasibility are central issues for any attempt to institutionalize population-level medical–bioethical mediation. Mediation requires trained facilitators, administrative support, stakeholder convening, documentation, monitoring, and time from participants. At population level, these requirements may be substantial, especially when conflicts involve multiple institutions, public authorities, and communities.

At the same time, unmanaged conflict can also be costly. It may generate litigation, repeated complaints, implementation delays, duplication of work, reputational damage, staff burnout, loss of public trust, and recurrent policy resistance. Evidence from medical mediation and alternative dispute resolution suggests that mediation can reduce the monetary and time costs associated with adversarial dispute resolution. In a recent preprint currently under journal review, Lioupi et al. [48] developed an open-access R/Shiny tool comparing medical mediation with litigation and projected substantial potential savings across high-, middle-, and low-income scenarios. To our knowledge, this is the first formal stochastic cost–benefit analysis for the benefits of medical mediation. Previous studies have reported practical benefits of medical mediation based on field experience and case-based evidence, but without formal quantitative projection. However, these estimates should not be directly generalized to population-level governance mediation. Rather, they are used here as preliminary evidence supporting the plausibility of economic evaluation in medical mediation, not as established evidence for population-level cost-effectiveness.

The proposed framework involves different actors, broader mandates, and additional infrastructure costs. Its economic value remains an empirical question. Future pilots should therefore include formal economic evaluation comparing the cost of mediation infrastructure with possible savings from reduced escalation, fewer repeated disputes, improved implementation adherence, shorter conflict cycles, fewer legal or administrative proceedings, and better institutional learning. Economic evaluation should be built into pilot design rather than treated as a later add-on.

### 7.4. Risks, Safeguards, and Limits of Use

Population-level medical–bioethical mediation also carries risks. Participation may become symbolic if key decisions have already been made or if the mediated process has no clear connection to implementation. Mediation may also create procedural delay when urgent action is required. Power asymmetries can allow stronger institutional actors to frame the conflict, select participants, or control the acceptable range of outcomes. Affected groups may be excluded if stakeholder mapping is narrow or politically convenient. Mediation may also be used to legitimize decisions that are essentially predetermined. Finally, an excessive preference for consensus may be inappropriate where authoritative action, legal adjudication, or emergency decision-making is required.

These risks do not invalidate the framework, but they show why safeguards are essential. Minimal safeguards should include a clear mandate, transparent criteria for activation, explicit stakeholder inclusion rules, conflict-of-interest declarations, protection against coercive participation, independent or arm’s-length mediation where needed, written documentation of agreements and disagreements, time limits in urgent settings, and defined links between mediated commitments and implementation. The process should also include monitoring and feedback so that mediation does not end with dialogue alone.

The framework should therefore be used selectively. It is most appropriate where ordinary governance remains formally active but no longer produces cooperation, legitimacy, or workable implementation. It is less appropriate where immediate emergency action is required, where legal rights must be adjudicated, where one party cannot participate safely or meaningfully, or where the sponsoring institution is unwilling to allow any genuine adjustment of position. In such cases, mediation may be premature, inappropriate, or ethically problematic.

The framework also has clear limitations. Population-level medical–bioethical mediation is not yet a mature institutional framework, and current evidence supports it mainly as a plausible conceptual extension rather than as a demonstrated system-level intervention. Institutional resistance, politicization, time and resource demands, and power asymmetries may all limit its use. New governance practices can disturb established routines, and adaptive governance depends on institutional fit rather than procedural aspiration alone [2,3,32]; a poorly designed mediation process could slow necessary decisions, reproduce domination, or give legitimacy to outcomes that have effectively already been decided. These limits are especially important in public-facing disputes, where participation can become symbolic if the mandate, decision pathway, or status of mediated commitments is unclear. The framework should therefore be read as a candidate governance approach, not as a general remedy for every contested health system decision.

### 7.5. Future Research and Evaluation

Future research should consolidate the conceptual boundary between clinical medical–bioethical mediation and population-level governance mediation. Existing clinical bioethics mediation work provides the anchor against which any extension should be tested [11,19,37]. Research should examine whether mechanisms associated with clinical mediation can travel to organizational and policy settings without losing their distinctive function. Implementation research is also needed on triggers, institutional location, role design, competencies, and training. Practice translation cannot be assumed from training alone [45,46]. Evaluation should examine legitimacy, trust, coordination, recurrence of conflict, learning effects, and costs across cases and institutional settings. Relevant assessment should look beyond agreement rates to institutional learning, resilience, and downstream effects [31,34,47]. Comparative case research would be useful. The appropriate institutional form is likely to vary across health systems, governance levels, and types of conflict. Pilot work could also test whether mediation changes implementation quality, stakeholder experience, and the durability of negotiated commitments.

### 7.6. Policy Implications

Policy implications follow from the same cautious logic. Mediation should be integrated into existing governance structures rather than built as an isolated add-on. Governance tools are most useful when embedded where conflict occurs, and institutional design shapes whether mediation is perceived as credible and usable [2,16,26,27,44]. Preparedness is another entry point. Systems under pressure need mechanisms that preserve coordination and legitimacy during disruption [29,33,35]. Policymakers can begin by defining minimal standards for mandate clarity, procedural fairness, independence, protection against capture, and monitoring, while recognizing that stronger empirical development is still required. The near-term task is not to mandate a uniform framework, but to identify where mediation capacity could be responsibly connected to public health authorities, hospital networks, regional governance bodies, ethics structures, patient safety programs, and complaint pathways. This would allow incremental development while maintaining caution about evidence, role definition, and institutional accountability.

## 8. Conclusions

Medical–bioethical mediation should be considered beyond the bedside as a possible governance innovation for health systems facing recurring ethical and institutional conflict. Its added value lies in bringing ethical clarification, stakeholder engagement, conflict transformation, negotiated implementation, and institutional learning into one process for governing disagreement. Claims for the framework should remain cautious. Population-level medical–bioethical mediation still requires empirical development, evaluation, and institutional testing. Its contribution should therefore be understood as a structured conceptual proposal whose value for sustainability, efficiency, and resilience remains to be tested through pilot implementation, comparative case research, and economic evaluation.

## Figures and Tables

**Table 1:** Distinguishing medical–bioethical mediation from adjacent approaches in healthcare conflict and governance.

Approach	Purpose and Level	Main Actors	Process and Output	Main Limitation
Clinical ethics consultation	Clarifies ethical issues in individual clinical cases and supports ethically defensible decision-making; primarily micro/clinical level.	Clinicians, patients, families, ethics consultants, institutional representatives.	Expert ethical analysis, advice, facilitation, and documentation; usually produces an ethics recommendation, chart note, or advice to the care team.	May clarify ethical principles without resolving relational conflict or negotiated implementation problems.
Bioethics mediation	Facilitates resolution of ethically charged clinical conflict through structured dialogue and shared problem-solving; primarily micro/clinical level.	Patients, families, clinicians, ethics mediators, institutional actors.	Neutral facilitation, ethical clarification, recognition of interests and values, and negotiated next steps; usually produces a mediated agreement or workable clinical plan.	Usually focused on individual care episodes rather than institutional or population-level governance.
Medical–bioethical mediation as proposed here	Governs ethically charged conflict across clinical, organizational, inter-institutional, and population-level settings; micro, meso, and macro levels.	Mediators, clinicians, managers, public health authorities, policy actors, community representatives, ethicists, legal advisors.	Structured conflict transformation combining stakeholder mapping, ethical clarification, mediated deliberation, negotiated commitments, implementation, and feedback; produces procedural/substantive commitments, implementation plans, monitoring, and learning.	Not yet a validated system-level intervention; requires institutional safeguards, resources, and empirical testing.
Public deliberation	Involves members of the public in reasoning about contested policy questions; population/policy level.	Citizens, community members, facilitators, policymakers, experts.	Inclusive public reasoning and deliberation about values and trade-offs; produces public recommendations, legitimacy input, or policy advice.	May generate public input without directly mediating conflict among responsible institutions or ensuring implementation.
Policy dialogue	Supports multi-stakeholder discussion of policy options and implementation challenges; policy/system level.	Policymakers, technical experts, stakeholders, civil society, implementers.	Structured dialogue around evidence, policy options, and implementation feasibility; produces policy learning, alignment, recommendations, or an implementation agenda.	May support coordination but does not necessarily address ethically charged conflict, mistrust, or relational breakdown.
Ombuds or complaint systems	Receives, investigates, and responds to grievances or complaints; clinical/organizational level.	Patients, families, staff, complaint officers, ombuds staff, institutions.	Complaint intake, investigation, advocacy, problem-solving, or referral; produces complaint resolution, explanation, apology, referral, or institutional response.	Often reactive and case-based; may not convene all stakeholders for mediated ethical deliberation.
Administrative governance	Makes, implements, and enforces organizational or policy decisions; organizational, regional, or national level.	Managers, regulators, public authorities, institutional leaders.	Formal authority, rules, mandates, accountability, and management processes; produces decisions, directives, regulations, or implementation plans.	May impose decisions without transforming conflict or rebuilding legitimacy among affected actors.
Conflict management frameworks	Identifies and reduces conflict through structured communication and escalation pathways; clinical/organizational level.	Healthcare staff, managers, patients, families, trained facilitators.	Early recognition, communication tools, escalation management, and prevention; produces reduced escalation, improved communication, or local resolution.	May focus on communication and de-escalation without explicit ethical analysis or population-level governance integration.

**Table 2:** Operationalizing the population-level medical–bioethical mediation framework.

Component	Purpose	Actors	Output	Possible Indicators
Conflict identification	Determine whether disagreement is ethically charged, recurrent, or implementation-limiting enough to require structured mediation.	Clinicians, managers, public health authorities, ethics committees, patient/community representatives, regional bodies.	Conflict trigger note, referral, or decision to assess suitability for mediation.	Recurrent disputes; escalation frequency; implementation blockage; complaints; litigation threats; stakeholder-reported conflict intensity.
Stakeholder mapping	Identify who is affected, who has authority or expertise, and who risks exclusion.	Mediator, convening body, institutional and community representatives, ethicists, legal/governance advisors.	Stakeholder map and inclusion plan.	Representation of affected/vulnerable groups; transparent selection criteria; power asymmetries identified.
Ethical issue clarification	Make explicit the values, rights, duties, harms, uncertainties, and distributional burdens involved.	Mediator, ethicist, clinicians, public health experts, legal advisors, affected parties/community representatives.	Ethical issue brief or shared statement of contested values and responsibilities.	Clarity of ethical questions; documented trade-offs; recognition of uncertainty; perceived fairness of issue framing.
Mediation and deliberation	Enable structured exchange of reasons, positions, interests, and possible movement.	Trained mediator, affected stakeholders, decision-makers, technical experts, community representatives.	Record of deliberation, clarified positions, and areas of agreement/disagreement.	Participation quality; perceived neutrality; satisfaction; trust; willingness to continue engagement; reduced escalation.
Negotiated solutions	Convert deliberation into workable substantive or procedural commitments.	Mediator, parties with implementation authority, affected stakeholders.	Agreement, procedural commitment, revised policy terms, monitoring arrangement, or documented non-agreement.	Clarity, feasibility, acceptability, role allocation, and degree of stakeholder endorsement.
Implementation	Ensure mediated commitments are translated into action rather than remaining symbolic.	Responsible institutions, managers, public health authorities, implementation teams, monitoring bodies.	Implementation plan with responsibilities, timeline, and review mechanism.	Adherence; delays; unresolved barriers; resource allocation; role clarity; corrective actions.
Feedback and learning	Capture lessons from the dispute and improve future governance capacity.	Convening body, mediator, quality/safety teams, governance bodies, ethics structures, public health agencies.	Learning report, revised procedure, training need, governance recommendation, or monitoring update.	Conflict recurrence; institutional learning; revised protocols; trust over time; reduced duplication; improved coordination.

**Table 3:** Illustrative applications of population-level medical–bioethical mediation.

Scenario	Conflict Problem	Mediation Function	Expected Governance Contribution
Vaccine hesitancy and mistrust	Institutional mistrust, safety concerns, contested communication	Structured engagement among communities, clinicians, and public health authorities	More credible handling of mistrust
Public health restrictions	Proportionality, liberty, protection, legitimacy	Ethical clarification and dialogue among policymakers and affected groups	Less escalation and clearer justification
Resource allocation	Scarcity, triage, benefit design, prioritization	Deliberation on fairness and workable procedural commitments	More legitimate allocation processes
Inter-hospital conflicts	Referral pathways, capacity, competition, coordination	Negotiation over roles, responsibilities, and monitoring	Better alignment across institutions
System reform conflicts	Political, organizational, and ethical disagreement	Structured conflict handling during redesign or restructuring	More governable reform implementation

## Data Availability

No new data were created or analyzed in this study. Data sharing is not applicable to this article.

## Abbreviations

The following abbreviations are used in this manuscript:

APHA	American Public Health Association
EU	European Union
